# Fast and Accurate Resonance Assignment of Small-to-Large Proteins by Combining Automated and Manual Approaches

**DOI:** 10.1371/journal.pcbi.1004022

**Published:** 2015-01-08

**Authors:** Markus Niklasson, Alexandra Ahlner, Cecilia Andresen, Joseph A. Marsh, Patrik Lundström

**Affiliations:** 1 Division of Biomolecular Technology, Department of Physics, Chemistry and Biology, Linköping University, Linköping, Sweden; 2 MRC Human Genetics Unit, MRC Institute of Genetics and Molecular Medicine, University of Edinburgh, Western General Hospital, Edinburgh, United Kingdom; University of Canterbury, New Zealand

## Abstract

The process of resonance assignment is fundamental to most NMR studies of protein structure and dynamics. Unfortunately, the manual assignment of residues is tedious and time-consuming, and can represent a significant bottleneck for further characterization. Furthermore, while automated approaches have been developed, they are often limited in their accuracy, particularly for larger proteins. Here, we address this by introducing the software COMPASS, which, by combining automated resonance assignment with manual intervention, is able to achieve accuracy approaching that from manual assignments at greatly accelerated speeds. Moreover, by including the option to compensate for isotope shift effects in deuterated proteins, COMPASS is far more accurate for larger proteins than existing automated methods. COMPASS is an open-source project licensed under GNU General Public License and is available for download from http://www.liu.se/forskning/foass/tidigare-foass/patrik-lundstrom/software?l=en. Source code and binaries for Linux, Mac OS X and Microsoft Windows are available.

This is a *PLOS Computational Biology* Software article.

## Introduction

Correct protein backbone assignments are pivotal for the interpretation of many NMR experiments and enable characterization of protein structure, dynamics and interactions at atomic resolution. Indeed, the backbone chemical shifts themselves have since long been recognized as faithful reporters of secondary structure [1]–[3] and more recently, methods to also calculate detailed protein tertiary structures [4]–[6] and to estimate protein flexibility [7] using chemical shifts as the sole restraints have emerged.

Resonance assignments are usually accomplished by recording a set of triple resonance experiments [8] that provides connectivities between spin systems in order to assign connected fragments to a particular region of the amino acid sequence based on chemical shift signatures. The process of assigning the backbone of a protein can however be cumbersome and the difficulty increases rapidly with increasing protein size. This is not only due to reduced sensitivity and more crowded spectra, but also because identical or similar amino acid sequence patterns likely appear more frequently, which requires identification of longer fragments for unambiguous assignment. An additional challenge is that the likelihood of chemical shifts that deviate significantly from consensus values increases [9].

Furthermore, in applications to larger proteins it is often necessary to deuterate aliphatic and aromatic positions to reduce relaxation losses and to maximize the benefit of the TROSY effect [10], [11]. Since the ^13^C-^2^H bond is slightly shorter than the ^13^C-^1^H bond [12], a side-effect of deuteration is isotope shifts for nuclei up to three covalent bonds from the attached deuteron. These can be substantial and to facilitate comparison with consensus chemical shift values it is crucial to compensate for the isotope effect.

The traditional approach of manually performing the assignments often leads to accurate results but may be very labor intensive. It is thus desirable to use automated routines, provided that they perform at a level comparable with the manual method. Different software that strive to accomplish this have been developed. For instance, AutoAssign [13], PINE Server [14], SAGA [15] and EZ-ASSIGN [16] are examples of programs designed for semi-automated or automated protein backbone assignment. A drawback with most automated routines is that the assignment process is either limited in terms of user control or is not intuitive enough for the average user.

To this end we have developed the interactive software COmputer-aided Matching and Peak ASSignment (COMPASS) with the intention of letting the user control the assignment process. In order to unambiguously assign connected spin systems, COMPASS identifies and analyzes fragments up to the size of ten residues. The likelihood for all possible assignments of such fragments is calculated and the most probable results are presented to the user who chooses among these suggestions. For deuterated proteins, isotope shifts are taken into account. Immediate feedback regarding the assignment process is provided and inconsistencies are easy to detect and address. User friendliness has been a key concern, with the aim of making the software intuitive to use. The outputs from COMPASS are assigned peak lists in Sparky format (T.D. Goddard and G.D. Kneller, SPARKY3, University of California, San Francisco), as well as the estimated secondary structure based on the secondary structure propensity (SSP) algorithm [17]. As we show below, COMPASS is very competitive for assigning proteins ranging in size from 60 to more than 300 residues.

## Design and Implementation

COMPASS is a standalone multi-platform application for Linux, Mac OS X and Microsoft Windows written in C++ and the graphical user interface (GUI) is developed with Qt 5.2.1. For graphical visualization of the calculated secondary structure, the Qt C++ widget QCustomPlot is used.

The required input for COMPASS is a file that specifies the amino acid sequence and peak lists for a selected set of NMR triple resonance experiments. A file that specifies the expected secondary structure is also accepted. The peaks must thus be picked manually or by using dedicated software [18], [19] and be referenced to 4,4-dimethyl-4-silapentane-1-sulfonic acid (DSS). The software can be subdivided into the modules ‘*Convert*’, ‘*Label*’, ‘*Analyze*’ and ‘*Assign*’. The ‘*Convert*’ module is a file converter that converts peak lists and protein sequence files to and from COMPASS format. The ‘*Label*’ module generates and labels spin systems from unassigned peak lists. Herein, a spin system is defined as all resonances that are in the same scalar coupled network as a particular amide proton. Thus, Cα(i-1), Cβ(i-1), CO(i-1), N(i), HN(i), Cα(i), Cβ(i) and CO(i) belong to the same spin system. Note that this implies that a particular ^13^Cα, ^13^Cβ or ^13^CO nucleus belongs to two different spin systems. The ‘*Analyze*’ module analyzes the spin systems for connectivities, generates fragments of connected spin systems and analyzes each fragment for the most probable assignments according to a chemical shift database. It also generates all necessary files needed for the ‘*Assign* ‘module where resonance assignments are performed and the secondary structure is calculated. Whereas ‘*Convert*’, ‘*Label*’ and ‘*Analyze*’ are fully automated, the ‘*Assign*’ module is interactive, i.e. the user has to approve suggestions. It also includes a fully manual mode where single spin systems can freely be assigned to any given position. If a COMPASS session is aborted prematurely, the status is saved so that the session can be reconvened at a later time point.

**Figure 1:**
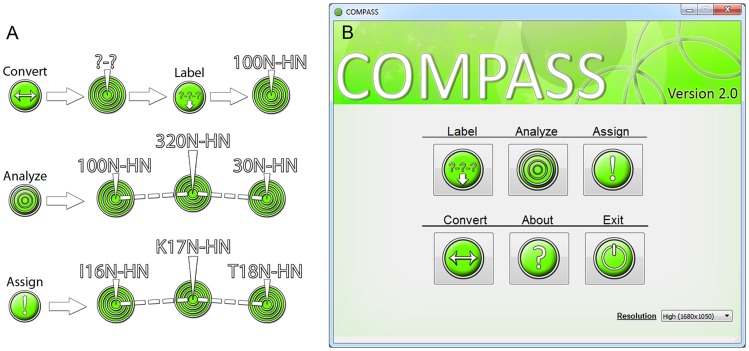
Schematic description of the four modules of COMPASS. (**A**) ‘Convert’ transforms provided peak lists and amino acid sequence files into COMPASS format. ‘Label’ assigns peaks to spin systems and labels them. ‘Analyze’ analyzes connectivities among spin systems and calculates the probabilities for all possible assignments for such fragments. ‘Assign’ is the interactive module where the user controls the assignment process, aided by the results from ‘Analyze’. (**B**) The COMPASS main menu provides easy access to the four modules.

The recommended process of using COMPASS, Fig. 1, can be described as follows:

1. Ensure that peak lists and other input data are in the correct format by using the ‘*Convert*’ module.
2. Generate arbitrarily assigned spin systems using the ‘*Label*’ module. Correct errors if needed.
3. Identify connectivities and calculate probabilities for the identity of generated fragments using the ‘*Analyze*’ module.
4. Assign the fragments using the ‘*Assign*’ module.
5. Fill in remaining gaps using the manual assignment mode of the ‘*Assign*’ module.

### Compass input format and the file conversion module

**Figure 2:**
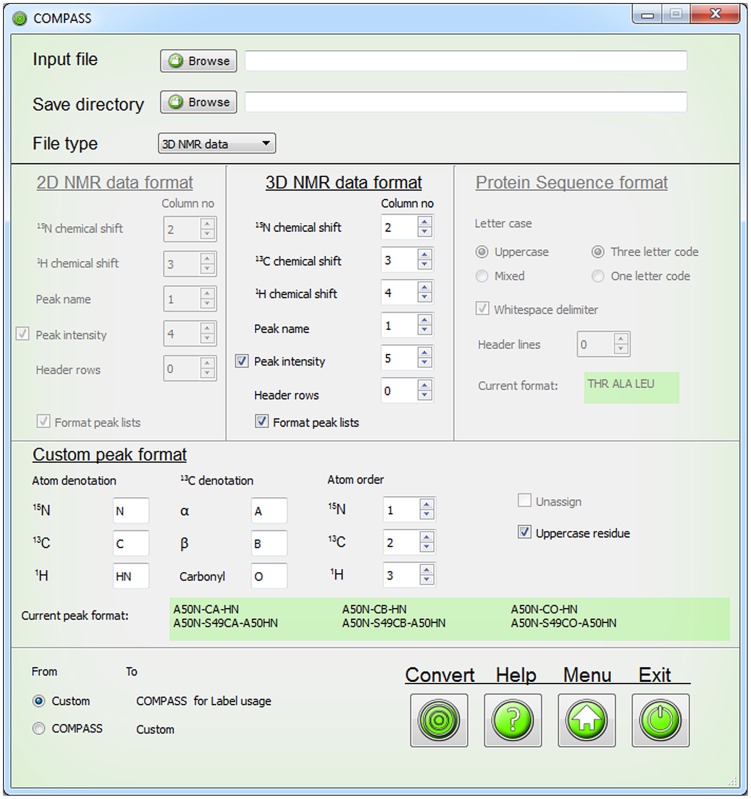
The COMPASS file conversion interface. The file converter enables conversion of peak lists and protein sequence files to formats compatible with COMPASS. The GUI instantly displays which format the protein sequence and peak assignments will be changed to or from.

To ensure that COMPASS is capable of performing analysis of submitted data it includes a file conversion module. Its interface is shown in Fig. 2. The user can submit the protein sequence and peak lists and convert the data from custom format to COMPASS format. The same module can be used to convert COMPASS output files back to custom format.

The internal format of COMPASS are peak lists in Sparky format and the supported types of peak lists are: HNCA(i)(i-1), HNCA(i-1), HNCB(i)(i-1), HNCB(i-1), HNCO(i)(i-1), HNCO(i-1) and ^15^N-^1^H HSQC. The ‘*i*’ and ‘*i-1*’ denotations refer to the ^13^C nuclei of internal and sequential residues, respectively. For HNCB lists, ^13^Cα chemical shifts in combination with ^13^Cβ chemical shifts are also accepted. For a description of which experiments that can be used to generate the different peak lists, see *S1 Table*.

A peak list in Sparky format consists of a header row and a blank row followed by entries with one peak per row. The first column of each entry is the name of the peak, the second column is the ^15^N chemical shift, the third column is a particular ^13^C chemical shift and the fourth column is the amide proton chemical shift in the case of peak lists for triple resonance experiments. For HSQC peaks lists the third column of course refers to the amide proton chemical shift. The ‘*Label*’ module requires an additional column that holds the peak intensities.

Unassigned peaks are named ‘*?-?-?*’ and assigned ones are named ‘*X(n)N-Cξ-HN*’ and ‘*X(n)N-Y(n-1)Cξ-X(n)HN*’ for peaks involving internal and sequential correlations to ^13^C nuclei, respectively. Here, *X* and *Y* denote the amino acid one letter code, *(n)* is the residue number or index for amide groups and internal correlations with carbons, *(n−1)* is the residue number or index for a sequential carbon correlation and *Cξ* is CA, CB or CO.

Since COMPASS also requires the protein sequence with the residues written in three-letter code and separated with whitespace, the ‘*Convert*’ module also converts a protein sequence written in various ways to this format and back to custom format.

**Figure 3:**
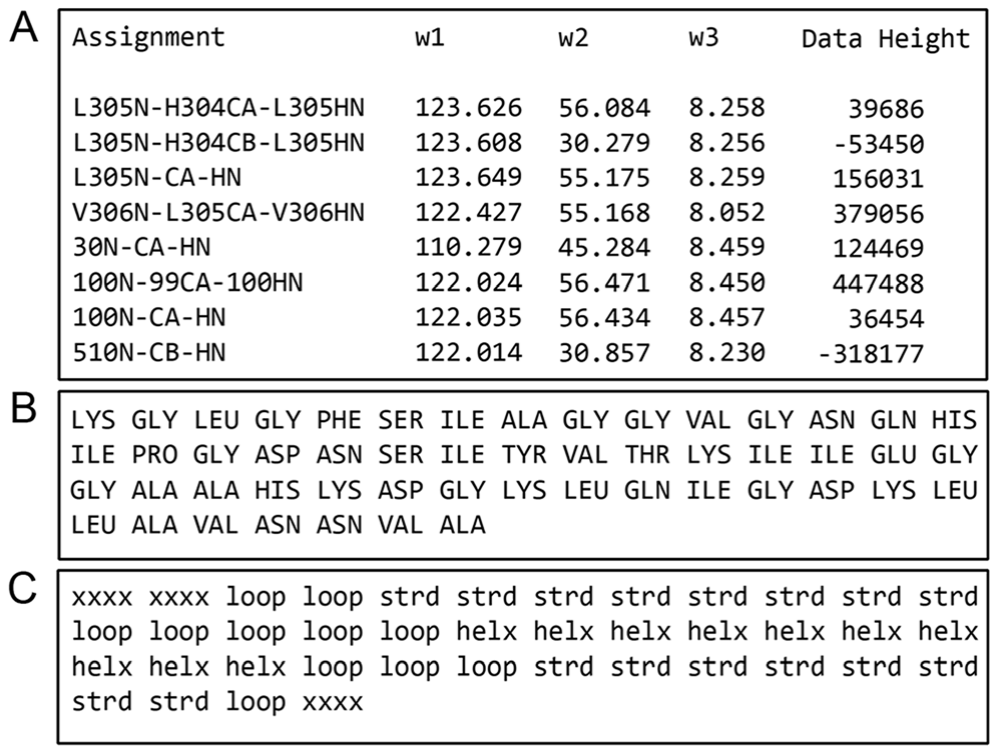
Examples of input files of the supported formats for COMPASS usage. (**A**) The HNCACB peak list file exported from Sparky with the column ‘Data Height’, indicating peak intensity. (**B**) The protein sequence file contains amino acids in uppercase three letter code separated with whitespace. (**C**) The secondary structure file must contain exactly one secondary structure element for each amino acid in the protein sequence file. The format is ‘xxxx’ for unknown secondary structure element, ‘helx’ for α-helix structure, ‘strd’ for β-strand structure and ‘loop’ for random coil structure written in lowercase letters and separated with whitespace.

Optionally, a file describing the protein secondary structure can be supplied to aid assignment. This file is ideally derived from the three-dimensional protein structure but secondary structure may also be predicted based on sequence homology. The format of this file is the secondary structure written as ‘*helx*’ for alpha helices, ‘*strd*’ for beta strands, ‘*loop*’ for random coil and ‘*xxxx*’ for segments of unknown structure. The words should be typed in lower case letters and be separated with whitespace. Note that ‘*Convert*’ cannot be used to convert to this format. Examples of input files in COMPASS format are shown in Fig. 3.

### Label module

**Figure 4:**
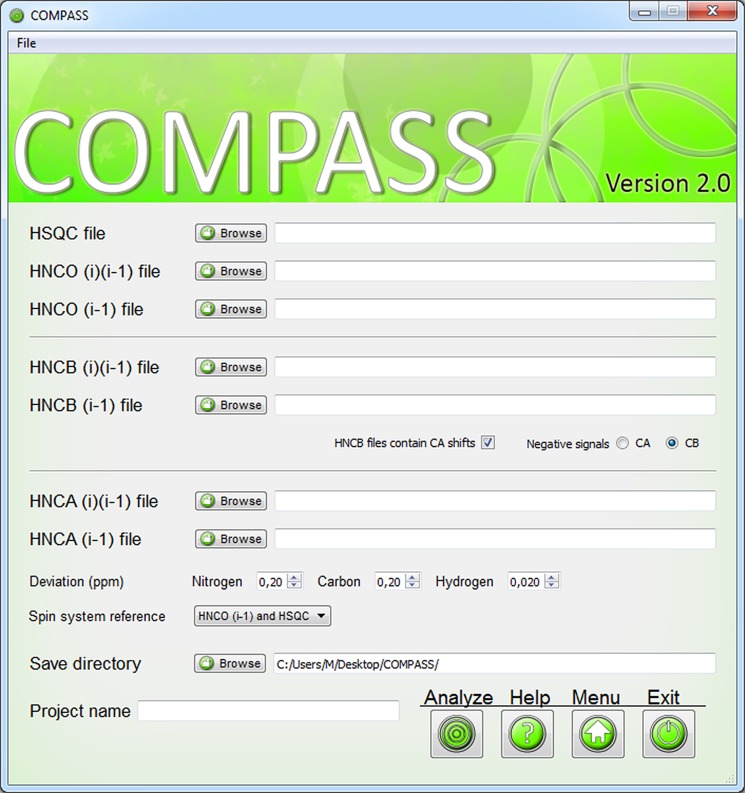
The user interface of the ‘Label’ module. Peak lists are submitted through separate dialogs and for HNCB(i)(i-1) data there is the option to include ^13^Cα shifts and select which of ^13^Cα or ^13^Cβ has negative peak intensity. The deviation boxes are used to tailor the spin system labeling to the studied protein and by iteratively altering these values the overlapping resonances may be more appropriately labeled. The spin system reference box specifies which peak list that will be used as reference for labeling of the spin systems.

The ‘*Label*’ module, Fig. 4, identifies spin systems in unassigned peak lists and labels them with arbitrary indices. The nuclei of two peaks are considered as one spin system if the chemical shifts of ^15^N and ^1^HN are equal within a user specified tolerance, termed ‘Deviation’. Using a large tolerance will ensure that all peaks in the spin system are labeled, but may cause overlapped peaks to be incorrectly assigned to the same spin system. If both (i-1) and (i)(i-1) peak lists are present, the peaks in the (i)(i-1) list are matched to the peaks in the (i-1) list to distinguish between sequential or internal correlations.

The input to the ‘*Label*’ module is peak lists supplemented with a column representing the peak intensity. The reasons for requiring the peak intensity are two-fold. For certain experiments, notably the HNCACB, correlations with ^13^Cα and ^13^Cβ are out of phase. Thus the sign of the intensity provides a convenient confirmation of whether a particular peak represents a correlation with ^13^Cα or^13^Cβ. Secondly, if peak lists from spectra that only include (i-1) correlations are missing, peak intensities can be used to reasonably reliably distinguish between internal and sequential correlations in (i)(i-1) peak lists.

We provide four options, termed ‘Spin system reference’, for assigning peaks to a spin system. If the ‘HSQC’ or ‘HNCO(i-1)’ option is selected, only peaks with ^15^N and ^1^HN chemical shifts matching peaks in these lists, respectively, are considered. The HSQC experiment is more sensitive than the HNCO(i-1), meaning that more potential spin systems can be found. However, it may be easier to distinguish overlapped peaks in the ^15^N and ^1^HN dimensions in the HNCO(i-1) spectrum. Alternatively, the peaks present in either spectrum can be used by the ‘HNCO(i-1) and HSQC’ option. If peaks are meticulously picked in all spectra, the option ‘All Lists’ may be used and will result in spin systems for all picked peaks. Successful generation of spin systems of course requires that all spectra are recorded at the same conditions and are referenced identically.

‘*Label*’ presents warnings for peaks that could not be assigned to a spin system. Because of the instantaneous execution the process may be iterated with different settings to optimize the number of successfully labeled peaks. The remaining warnings must be addressed manually since the ‘*Analyze*’ module cannot continue using invalid data. A parameter file is generated that may be used to rerun ‘*Label*’ or when the output of ‘*Label*’ is analyzed with ‘*Analyze*’.

### Analyze module

**Figure 5:**
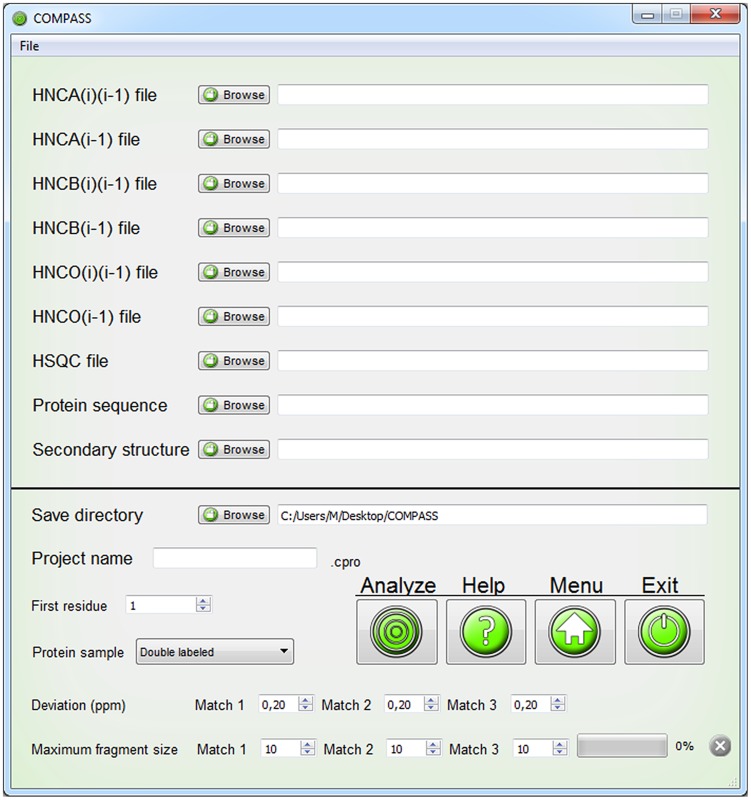
User interface for the ‘Analyze’ module. Input files are imported through separate file browser dialogs. The HNCB(i) and HNCB(i)(i-1) may also include ^13^Cα chemical shifts. The user must select a save directory and a project name. Several additional parameters may be adjusted.

The purpose of ‘*Analyze*’ is to analyze spin systems for connectivities, to generate fragments of connected spin systems and to score the fragments for probability of belonging to a certain region of the amino acid sequence. The ‘*Analyze*’ interface is shown in Fig. 5.

Spin systems are connected to generate fragments by matching one, two or three (if available) of the ^13^Cα, ^13^Cβ and ^13^CO chemical shifts from different spin systems according to user set tolerances. These different ways of connecting spin systems are called ‘*Match 1*’, *Match 2*’ and *Match 3*’, respectively. Glycine residues of course lack ^13^Cβ nuclei but are readily identified and are also included in fragments generated by ‘*Match 3*’. Results obtained with ‘*Match 3*’ are naturally most reliable but also ‘*Match 2*’ and even ‘*Match 1*’ results are valuable for establishing spin system connectivities when one or two of the ^13^Cα, ^13^Cβ and ^13^CO chemical shifts for a spin system are missing due to sensitivity or overlap issues. Generation of long fragments with ‘*Match 2*’ and especially ‘*Match 1*’ however leads to error prone results and should be avoided.

COMPASS generates a maximum of 1000 fragments of up to ten residues by extending connected spin systems with another spin system if possible. If the limit of 1000 generated fragments is reached, the analysis is restarted using decreased tolerances to optimize the quality of the analysis as well as to reduce the runtime. To further optimize the runtime of the analysis, the user may limit the maximum fragment size to a value smaller than ten.

The ^13^C chemical shifts of the fragments are scored according to similarity with consensus values from the RefDB database [20] for ^13^C chemical shifts of all possible fragments of the same size of the provided amino acid sequence. The score is represented as a reduced χ^2^ value where a low value indicates a higher probability for a particular assignment.
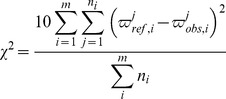
(1)In Eq. 1, the outer summation runs over the number of residues in the fragment, the inner summation runs over the number of chemical shifts that are matched with the database values for residue *i*, 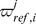 is the RefDB value for nucleus *j* of residue *i* and 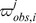 is the corresponding observed chemical shift. The index *j* refers to ^13^Cα, ^13^Cβ or ^13^CO, the denominator is the total number of chemical shifts that are analyzed and the factor of ten is a scaling factor to change the range of χ^2^. Since the ^13^Cα, ^13^Cβ and ^13^CO chemical shifts are highly dependent on the dihedral angles [1], the calculation is performed for all possible combinations of secondary structure for the residues in the fragment.

For deuterated proteins, isotope shifts are compensated for before calculating *χ* ^2^. We estimated the isotope shifts for ^13^Cα, ^13^Cβ or ^13^CO of the twenty amino acids by comparing spectra recorded for protonated and perdeuterated thioredoxin domain of human Grx3 [21]. The values used for compensation can be found in *S2 Table* and correlate well with values determined for α-synuclein [12] but are consistently lower than ones calculated using the method of Venters et al. [22]. We also determined isotope shifts for partially deuterated proteins in the same way. However, since the values were very similar to the ones for perdeuterated protein and were less precisely determined, we chose to compensate for isotope shifts in partially deuterated proteins in the same way. No attempt is made to take the dihedral angle dependence of isotope shifts [23] into account.

The primary output of the ‘*Analyze*’ is files that hold the 20 most probable assignments for each fragment. Each proposed assignment is given a unique index. Separate files are generated for different fragment sizes and different values of ‘*Match*’.

### Assign module

**Figure 6:**
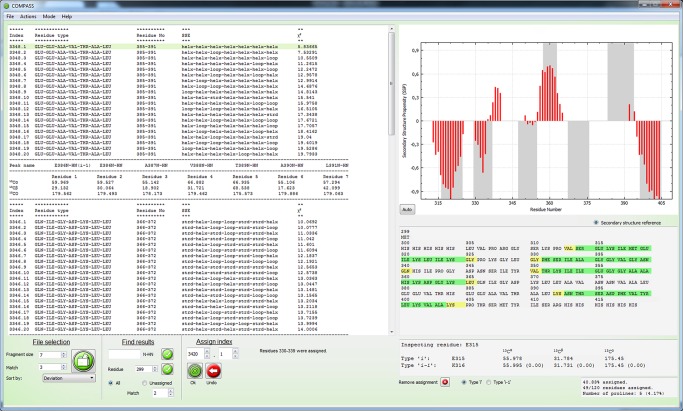
The user interface for the ‘Assign’ module. The browser to the left displays the result of ‘Analyze’. Below the browser there are options to change which file is viewed and how to scan the lists for specific results. Next to these there are tools for assigning the spin systems according to aforementioned indices. Completed assignments are displayed in the interactive protein sequence to the right. Information regarding chemical shifts of assigned spin systems is displayed below the protein sequence and at the bottom right statistics are displayed. The calculated secondary structure is displayed above the protein sequence and is updated when a new fragment is assigned. The menu bar allows for further operations, including displaying warnings and enabling the ’Manual Assignment mode’.

In the *Assign*’ module the results from ‘*Analyze*’ are used to perform backbone assignments. Its interface, Fig. 6, consists of a file browser that displays a selected ‘*Analyze*’ result file, an interactive protein sequence where the progress is displayed as well as a graphical representation of the calculated secondary structure. Information, such as chemical shifts and original names of assigned peaks, as well as statistics regarding backbone assignment completeness, is displayed below the protein sequence.

The displayed result sets consist of the 20 most probable assignments with a proposed secondary structure and the reduced χ^2^ value. If a file with the expected secondary structure has been submitted, any result that perfectly matches it is highlighted. The result files can be sorted according to the highest probability of the results or according to the proposed position in the amino acid sequence. A useful way of using the ‘*Assign*’ function is to start analyzing files with large fragment sizes and high ‘*Match*’ values, as these results are based on more information and can be used to assign large portions of the protein sequence quickly and unambiguously. Their reduced χ^2^ values are often very indicative of correct or incorrect assignments, since larger fragments often are unique in most amino acid sequences.

The assignment of a fragment is performed by either clicking a corresponding index directly in the file browser, or by entering the index in a spin box and verifying the selection. Completed assignments continuously yield updated peak lists, updated information regarding the progress and an updated calculation of the secondary structure. The updated information includes an amino acid sequence that is highlighted in green for assigned residues corresponding to spin systems that are linked via matching ^13^C chemical shifts to sequential neighbors, and in yellow for assigned residues that are not. The amino acid sequence is interactive and when a residue is clicked, chemical shift information of connected spin systems is displayed. Updated statistics regarding the completeness are also provided.

**Figure 7:**
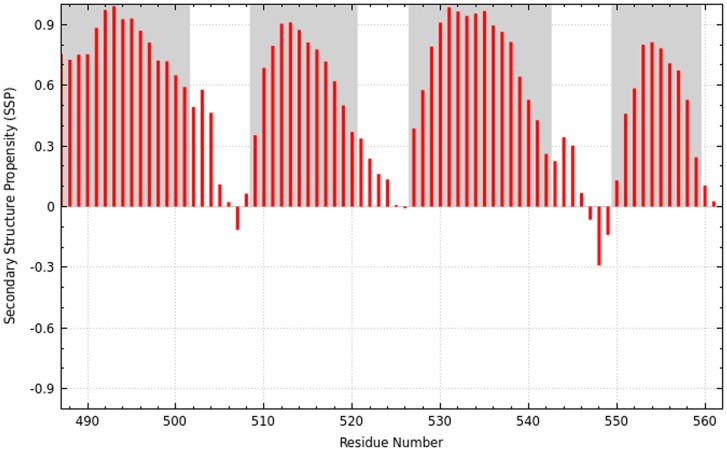
Secondary structure for the CLD(B) domain from CDPK3 based on COMPASS assignments. Red bars represent the calculated SSP score [17] for each residue and gray areas indicate values from the submitted secondary structure file, in this case based on the crystal structure of a larger fragment of the protein [33]. Positive and negative values represent α-helix and β-strand propensity, respectively.

Knowledge of protein secondary structure is both valuable on its own, and can be a very useful aid in the assignment process and for validation of resonance assignments. Since the secondary structure can be accurately predicted if backbone chemical shifts are known, it is recalculated and displayed as soon as a new fragment has been assigned using the SSP algorithm [17]. The SSP score for a residue ranges from approximately −1 for a fully developed β-strand to approximately 1 for a fully developed α-helix. It is calculated by weighted averaging over five residues so that also the chemical shifts of two residues prior to and after the analyzed residue are taken into account, which results in smoothening. The SSP scores are plotted against residue number in the GUI. If the expected secondary structure is provided, it is possible to highlight it in the SSP graph. The calculated secondary structure may be exported as an image as shown in Fig. 7.

While assigning the protein backbone, the results lists will shrink in size and eventually comprise lists with single spin systems. The assignment of these fragments is aided by displaying information of eligible positions in the protein sequence to find out where they can be accommodated. For this purpose, ‘*Assign*’ features a function that allows the user to display possible assignments for a submitted residue number. The user can select if the function should scan complete result lists or the result lists that contain the remaining unassigned residues.

If the user notices errors in peak picking while using ‘Assign’, there is a function included to immediately correct such errors. Selecting ‘Actions>Edit chemical shifts’ will open a dialogue where the chemical shifts of ^13^Cα, ^13^Cβ and ^13^CO may be changed or added for each existing spin system. These changes will not affect the result lists from the analysis but will be used to correctly update the SSP analysis as well as the output files. This eliminates the extra tasks of performing manual changes after COMPASS usage and repeating the analysis with updated lists to yield a correct SSP analysis.

As analysis of single spin systems may fail, COMPASS features a function to manually assign spin systems to any position. By selecting ‘*Mode>Manual Assignment mode*’, from the menu bar, the GUI is replaced with a similar interface that enables manual assignment as well as calculation of χ^2^ to facilitate comparison of manual assignments with the results from the automated analysis. This mode is useful for assigning spin systems that have aliased peaks or other errors associated with peak picking that the user may detect during assignment. The fact that some spin systems can be assigned that give ambiguous results in the automated analysis also motivates the inclusion of a manual mode. At any time the user can switch between the semi-automated and manual mode, but we recommend avoiding manual assignments for as long as possible, since they lack the reliability of the COMPASS automated analysis. It is however possible to generate warnings when assigned spin systems or fragments do not fit together. Using the strategies described above, COMPASS can quickly assign protein backbone resonances to high completeness and an accuracy that rivals that of fully manual methods.

## Results

**Figure 8:**
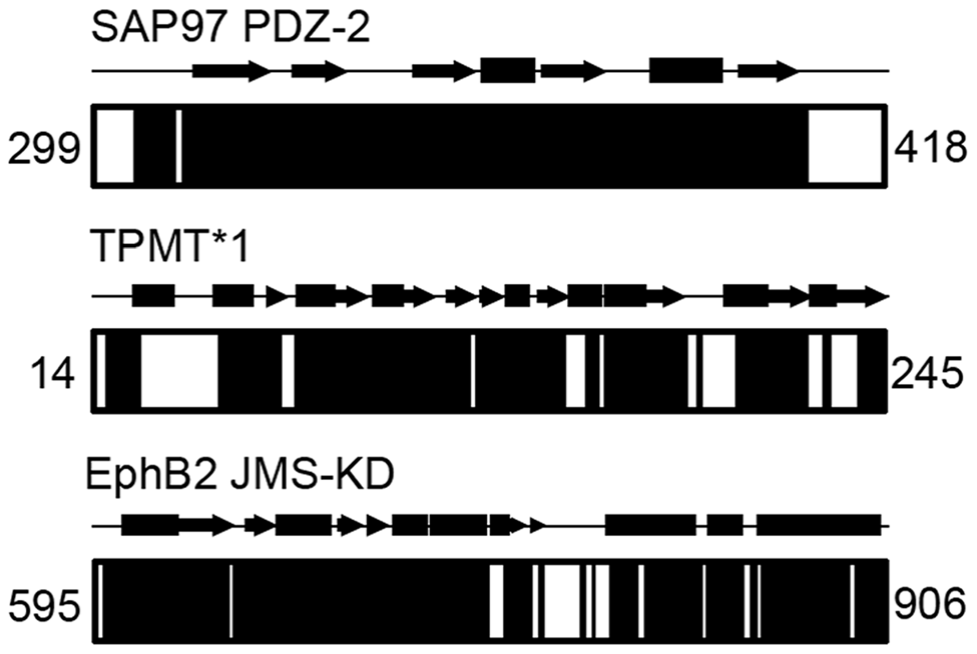
Assignment completeness for the proteins SAP97 PDZ-2, TPMT*1 and EphB2 JMS-KD. Solid black areas represent assigned regions. The secondary structure as determined from the crystal structures [30], [31], [34] with α-helices and β-strands represented as rectangles and arrows, respectively, are shown above the diagrams. The numbers refer to the residue numbers of the N-terminal and C-terminal residues.

The performance of COMPASS was evaluated by using it to assign the backbone of six proteins with different characteristics. The proteins were Abp1p SH3 domain [24], E140Q Tr2C [25], CDPK3 CLD(B) [26], SAP97 PDZ-2 [27], TPMT*1 [28] and EphB2 JMS-KD [29] that differ in size as well as secondary structure composition. More details about the proteins, sample conditions and NMR experiments can be found in *Supporting Information*. The completeness and accuracy of the assignments were primarily gauged by comparison with careful manual analysis. By completeness we mean two different things: 1) the fraction of peaks in the submitted peak lists that could be assigned and 2) the fraction of assigned residues of the protein. Completeness according to both these definitions is presented in Table 1, where Peaks refers to the percentage of assigned peaks in the supplied peak lists and NH refers to the percentage of assigned amide groups in the proteins. For four small proteins of 59–120 residues, the completeness was 91%–100% regarding fraction of assigned peaks and for all proteins essentially all visible backbone amide groups could be assigned. The low number of 82.6% assigned backbone amide groups for SAP97 PDZ-2 is due to the presence of N-terminal and C-terminal histidine tags included in the calculation. As shown in Fig. 8, all backbone amides except ones from these unstructured tails could be assigned. The assignments were almost as or as complete as ones obtained by manual analysis and we did not detect any assignment inconsistencies between the two approaches. We also tested COMPASS for more challenging systems involving larger proteins and incomplete data sets. The protein TPMT*1 comprises 232 residues and yields spectra where a large fraction of the peaks are broadened beyond detection. The percentage of peaks that could be assigned was 80% and the percentage of assigned backbone amide groups was 63%. Once again, this may sound low but from Fig. 8 it is obvious that unassigned residues cluster to specific regions. We analyzed the structural context of these regions and with few exceptions they mapped to the active site and cofactor binding regions of the protein [30], suggesting that these are highly flexible and broadened beyond detection due to rapid amide proton exchange with solvent. This is not surprising considering the relatively high pH (7.3) and the fact that the crystal structure suggests these regions are largely unstructured. A time-consuming manual analysis only improved the percentage of assigned backbone amides to 68%, again showing that COMPASS almost yields as complete assignments as manual methods. We also assigned the 312-residue EphB2 JMS-KD to check performance for larger, deuterated proteins. With the aid of COMPASS we were able to assign 94.6% of the peaks and 79.0% of the backbone amide groups, where unassigned residues largely were confined to the activation segment that is invisible in the crystal structure of the protein [31]. As for TPMT*1, manual analysis only improved the results marginally. We found no bias in success rate towards α-helical or β-strand structural elements.

**Table 1:** Completeness of assignments performed with COMPASS and manual methods.

Protein	Residues	Secondary structure	COMPASS		Manual analysis	
		α (%),β (%), r.c. (%)	Peaks (%)^1^	NH (%)^2^	Peaks (%)	NH (%)
Abp1p SH3	59	0, 40, 60	99.1	98.3	99.1	98.3
E140Q-Tr2C	73	51, 8, 41	99.4	95.9	100	97.3
CDPK3 CLD(B)	76	72, 0, 28	90.8^3^	90.8^3^	90.8^3^	90.8^3^
SAP97 PDZ-2	120	12, 32, 45	100	82.6	100	82.6
TPMT*1	232	39, 27, 34	80.2	62.9	84.9	67.7
EphB2 JMS-KD	312	47, 12, 41	94.6	79.0	96.6	81.7

1 Percentage of peaks in all peak lists that were assigned.

2 Percentage of backbone amide groups that were assigned.

3 The figures are equal by coincidence.

We have not tested COMPASS for proteins larger than EphB2 JMS-KD but we are confident that the methodology can be applied to systems with at least twice as many residues following minor modifications to the software. These include adding support for peak lists from four-dimensional experiments, such as the 4D HNCACO and 4D HNCOCA experiments that were used to assign the backbone of malate synthase G (723 residues) [32]. It is also possible to include the capability of utilizing peak lists from NOESY experiments to resolve ambiguities. These modifications could also increase the assignment completeness for smaller proteins.

Although we did not detect any inconsistencies between assignments performed using COMPASS and manual methods, this does not prove that the assignments are correct. To conclusively determine the accuracy of COMPASS, we generated synthetic peak lists for a 76 residue protein with *known* chemical shifts. We used the amino acid sequence but not necessarily the actual chemical shifts of CLD(B) of CDPK3 and denote this protein CDPK3 CLD(B)^HYP^. To simulate the experimental setting, Gaussian distributed noise was added to the chemical shifts in the generated peak lists and we repeated the analysis using data sets with randomly omitted peaks so that the investigated peak lists had 100%, 90%, 80%, 70% of the original data set available for analysis. The results of this analysis are displayed in Table 2. It is reassuring to see that the assignments were indeed 100% correct for the full data set and that surprisingly good results were obtained also for data sets with a large fraction of missing peaks. For the 90% complete data set it was also possible to assign all peaks correctly although lack of information meant that only 59 of the 76 backbone amide groups could be assigned. Even for a data set that only retained 70% of the peaks it was possible to unambiguously assign 81% of the peaks and almost half of the backbone amide groups.

**Table 2:** Fraction of correctly assigned peaks for CDPK3 CLD(B)^HYP^.

Peaks available for assignment	Peaks (%)^1^	NH (%)^2^
100%	100	98.7
90%	100	77.6
80%	90.8	59.2
70%	81.0	39.5

1 Percentage of peaks in all peak lists that were assigned.

2 Percentage of backbone amide groups that were assigned.

Since a COMPASS session comprises automated as well as manual steps, it is not possible to precisely state the time needed for completing the assignments. Contrary to most other assignment programs COMPASS includes checkpoints for the consistency of the input data and erroneous data must be corrected manually before proceeding. For instance, after initial peak picking and conversion of file formats, the ‘*Label*’ module and manual correction are used iteratively until the data is consistent. Whereas the ‘*Label*’ function itself is instantaneous, the time required for resolving inconsistencies depends on how carefully the peaks were picked, the size of the protein and complexity of the spectra. For small proteins with well-resolved spectra, the process is usually completed within an hour, whereas larger or unfolded proteins may require several hours. The performance of the ‘*Analyze*’ module is dependent on protein size, completeness of data and settings in COMPASS. Higher tolerances will increase the time for analysis, but also yield larger result sets. In all cases hitherto tested, the time for ‘*Analyze*’ ranges from several minutes to several hours. During this time, no user input is required. In the final step of COMPASS, ‘*Assign*’, the user reviews and approves suggestions for assignments. The time spent assigning the backbone highly depends on the results from the analysis and the available information in the analyzed spectra. In our hands, small proteins (<100 residues) are assigned as completely as the input data allows within an hour. Naturally, larger proteins require more time and how much depends on the aforementioned parameters. Comparing the runtime of COMPASS, including the manual steps, with that of manual assignment, there is a tremendous difference in favor of COMPASS.

The performance of COMPASS was compared to that of AutoAssign [13], PINE Server [14], SAGA [15] and EZ-ASSIGN [16]. AutoAssign is a web-based tool that accepts data in the form of unassigned peak lists and generates assigned peak lists. PINE Server is also web-based and in addition to assigned peak lists, it also calculates the secondary structure. SAGA is a standalone application that generates a list with possible resonance assignments to be used as a compliment when assigning a protein manually. EZ-ASSIGN is a novel program that has been reported to outperform AutoAssign, PINE Server and SAGA [16]. It generates lists with possible assignments and calculated probabilities and allows extensive customization prior to analysis.

The different types of software were compared using identical input data and similar settings for three proteins with different properties. PDZ-2, comprising 120 residues, was chosen as an example of a small, well-behaved protein. TPMT*1, comprising 232 residues, was used as an example of a medium-sized protein with an incomplete data set. Lastly, EphB2 JMS-KD, comprising 312 residues, was selected as an example of a large deuterated protein. The input data consisted of HNCA(i)(i-1), HNCA(i-1), HNCB(i)(i-1), HNCB(i-1), HNCO(i)(i-1), HNCO(i-1) peak lists. ^15^N-^1^H HSQC peak lists were also included if supported by the software. To make the comparisons as fair as possible, no manual assignments were made afterwards although COMPASS includes this capability. The results are presented in Table 3.

**Table 3:** Comparison of assignment completeness using different software.

Protein	COMPASS		AutoAssign		EZ-ASSIGN		PINE Server		SAGA	
	Peaks (%)^1^	NH (%)^2^	Peaks (%)	NH (%)	Peaks (%)	NH (%)	Peaks (%)	NH (%)	Peaks (%)	NH (%)
SAP97 PDZ-2	100	82.6	83.3	75.6	91.2	77.4	69.0	74.8	88.8	74.8
TPMT*1	80.2	62.9	18.0	12.9	44.7	33.2	64.4	49.6	25.6	19.8
EphB2 JMS-KD	94.6	79.0	17.5	24.7	67.6	57.3	84.6	74.9	37.8	31.5

1 Percentage of peaks in all peak lists that were assigned.

2 Percentage of backbone amide groups that were assigned.

COMPASS had the highest completion rates of the compared software in all investigated cases. For the PDZ domain all software performed reasonable well, although notably PINE Server suffered from difficulties assigning the HNCA(i)(i-1) peak list. For TPMT*1 the results were significantly poorer, likely reflecting problems handling incomplete data sets. No other software was able to assign more than 65% of the peaks and the best software in terms of fraction of assigned backbone amides, PINE Server, was only able to assign 50%, which is 13 percent points below COMPASS. The completion level according to this criterion for the other software ranged between 13% and 33%. For assignment of JMS-KD, there was a large difference between the performances of the different software. Once again, PINE Server performed best and assigned 85% of the peaks and 75% of the amide groups compared to 95% and 79%, respectively, for COMPASS. EASY-ASSIGN managed to assign 57% of the amide groups whereas AutoAssign and SAGA managed 25% and 32%, respectively.

Since COMPASS was developed for applications to both small and large proteins, and it is the only software that is able to compensate for isotope effects, it is perhaps not surprising that it performs better for larger proteins. It is also quite possible that the performance of PINE Server and AutoAssign would be better if data from the additional experiments that these software support were included and that SAGA and EZ-ASSIGN might perform better if other settings of the input parameters were used. The results should therefore be interpreted with some caution.

In terms of runtime it is difficult to compare the programs since the results are presented differently. Whereas PINE Server and AutoAssign present the user with files ready for import into software such as Sparky, SAGA and EZ-ASSIGN require additional work for constructing the final peak lists. COMPASS is most straightforwardly compared to the former two and as expected has a considerably longer runtime. However, overall time is likely saved considering the need for completing the assignments to the level of COMPASS results when using other software.

In conclusion COMPASS is a user-friendly software that enables the accuracy of manual assignment at a fraction of the time. The greatest advantages include user-control of the assignment process, instant graphical feedback on the progress and calculation of the secondary structure using a robust algorithm. The runtime of COMPASS is longer than that of fully automated software but we are convinced that the superior performance in terms of accuracy and completeness makes up for this and that COMPASS should be a valuable tool for backbone resonance assignments of proteins.

## Availability and Future Directions

COMPASS is an open-source project licensed under GNU General Public License and is available for download from http://www.liu.se/forskning/foass/tidigare-foass/patrik-lundstrom/software?l=en. Source code as well as binaries for Linux, Mac OS X and Microsoft Windows is available. At the same web site users can sign up to a mailing list where information, improvements and new versions of COMPASS will be announced.

Users may extend the functionality of COMPASS by modifying the source code. An example of a modification that we would welcome is improved performance for the assignment of intrinsically disordered and proline rich proteins by adding support for peak lists derived from experiments that provide both internal and sequential correlations for proline residues. Another example is adding increased functionality to the file conversion module. Our top priority for modifications is improvement of the algorithm that scores the connected spin systems in order to further increase speed and accuracy. We also plan to add support for peak lists from additional experiments that will increase the assignment completeness for small and medium-sized proteins as well as extend the limit on how large proteins that can be assigned using COMPASS.

## Supporting Information

S1 Table Description of the peak lists supported by COMPASS and the NMR experiments they can be derived from.
(DOC)

S2 Table Isotope shift compensation for deuterated protein samples. Values used to compensate ^13^Cα, ^13^Cβ and ^13^CO chemical shifts are shown.
(DOC)

S1 Text Isotopic labeling schemes, protein concentrations and buffer conditions as well as magnetic field strengths and temperatures used for the NMR experiments are described. The various experiments used to assign the backbone of the proteins are also listed.
(DOC)
