# Supplementary material for: Fast and Accurate Resonance Assignment of Small-to-Large Proteins by Combining Automated and Manual Approaches
Source: PLoS Comput Biol. 2015 Jan 8;11(1):e1004022. doi: 10.1371/journal.pcbi.1004022 (PMC4288728; doi:10.1371/journal.pcbi.1004022)
Supplement: S1 Table — Description of the peak lists supported by COMPASS and the NMR experiments they can be derived from. (DOC) [file pcbi.1004022.s001.doc]

**Table S1. Peak lists for COMPASS and NMR experiments they can be derived from.**

| **Peak list** | **Experiment** |
| --- | --- |
| HNCA(i)(i-1) | HNCA, iHNCA |
| HNCA(i-1) | HN(CO)CA |
| HNCO(i)(i-1) | HN(CA)CO |
| HNCO(i-1) | HNCO |
| HNCB(i)(i-1) | HNCACB, HN(CA)CB, iHNCACB, iHN(CA)CB |
| HNCB(i-1) | CBCA(CO)NH, HN(CO)CACB, HN(COCA)CB |
