# Supplementary material for: Fast and Accurate Resonance Assignment of Small-to-Large Proteins by Combining Automated and Manual Approaches
Source: PLoS Comput Biol. 2015 Jan 8;11(1):e1004022. doi: 10.1371/journal.pcbi.1004022 (PMC4288728; doi:10.1371/journal.pcbi.1004022)
Supplement: S2 Table — Isotope shift compensation for deuterated protein samples. Values used to compensate 13Cα, 13Cβ and 13CO chemical shifts are shown. (DOC) [file pcbi.1004022.s002.doc]

**Table S2. Isotope shift compensation^1^ for deuterated^2^ proteins.**

| **Residue** | **∆δ ^13^C^α^ (ppm)** | **∆δ ^13^C^β^ (ppm)** | **∆δ ^13^C^O^ (ppm)** |
| --- | --- | --- | --- |
| Ala | 0.45 (18^2^) | 0.82 (18) | -0.03 (19) |
| Arg | 0.45 (3) | 0.98 (3) | -0.02 (3) |
| Asn | 0.28 (2) | 0.58 (2) | -0.02 (2) |
| Asp | 0.35 (2) | 0.61 (2) | -0.03 (2) |
| Cys | 0.62 (1) | 0.66 (1) | 0.02 (1) |
| Gln | 0.46 (5) | 0.85 (4) | -0.05 (5) |
| Glu | 0.47 (14) | 0.87 (14) | -0.03 (14) |
| Gly | 0.42 (6) | n.a. | -0.01 (6) |
| His | 0.42 (4) | 0.89 (4) | 0.03 (4) |
| Ile | 0.42 (3) | 0.94 (2) | -0.03 (2) |
| Leu | 0.42 (10) | 1.10 (11) | -0.04 (11) |
| Lys | 0.44 (9) | 1.02 (9) | -0.05 (9) |
| Met | 0.46 (2) | 0.86 (2) | -0.03 (2) |
| Phe | 0.35 (6) | 0.86 (6) | -0.01 (5) |
| Pro | 0.42 (3) | 0.85 (3) | -0.06 (6) |
| Ser | 0.36 (8) | 0.72 (6) | -0.01 (9) |
| Thr | 0.38 (2) | 0.55 (2) | -0.02 (2) |
| Trp | 0.34 (2) | 0.67 (2) | -0.03 (2) |
| Tyr | 0.40 (1) | 0.89 (1) | -0.05 (1) |
| Val | 0.46 (12) | 0.94 (12) | -0.04 (1) |

^1^Defined as the value added to the ^13^Cα, ^13^Cβ and ^13^CO chemical shifts of the analyzed spin system when calculating the *χ^2^*value for a given amino acid. ^2^The same compensation is applied for both perdeuterated and partially deuterated proteins. ^3^Number of chemical shifts used in the calculation. ^3^Number of chemical shifts used in the calculation.
