# Supplementary material for: Fast and Accurate Resonance Assignment of Small-to-Large Proteins by Combining Automated and Manual Approaches
Source: PLoS Comput Biol. 2015 Jan 8;11(1):e1004022. doi: 10.1371/journal.pcbi.1004022 (PMC4288728; doi:10.1371/journal.pcbi.1004022)
Supplement: S1 Text — Isotopic labeling schemes, protein concentrations and buffer conditions as well as magnetic field strengths and temperatures used for the NMR experiments are described. The various experiments used to assign the backbone of the proteins are also listed. (DOC) [file pcbi.1004022.s003.doc]

## Protein samples and NMR spectroscopy

All NMR data was acquired at Varian Inova spectrometers operating at proton Larmor frequencies of 600 MHz or 800 MHz.

Data for ^13^C/^15^N labeled second PDZ domain from human SAP-97 (SAP-97 PDZ-2) was recorded at 800 MHz, 25°C and consisted of HNCACB, CBCA(CO)NH, HN(CA)CO, HNCO and HSQC experiments. Sample conditions were 0.3 mM protein, 50 mM KP_i_, pH 6.9, 10% D_2_O.

Data for ^13^C/^15^N labeled *S. cerevisiae* Abp1p SH3 domain was recorded at 600 MHz, 25 °C and consisted of HNCACB, HN(CA)CO and HSQC experiments. Sample conditions were 0.5 mM protein, 50 mM NaP_i_ pH 7.0, 100 mM NaCl, 1 mM EDTA, 200 μM NaN_3_, 10% D_2_O.

Data for ^13^C/^15^N labeled E140Q mutant of tryptic fragment 2 of bovine calmodulin (E140Q-Tr2C) was recorded at 600 MHz, 35 °C and consisted of HNCACB, HN(CA)CO and HSQC experiments. Sample conditions were 0.7 mM protein, 20 mM CaCl_2_, 200 μM NaN_3_, 10% D_2_O, pH 6.0.

Data for ^13^C/^15^N labeled second calcium ligating domain of *P. falciparum* calcium dependent kinase 3 (CDPK3 CLD(B)) were recorded at 600 MHz, 25 °C and consisted of HNCACB, CBCA(CO)NH, HN(CA)CO, HNCO and HSQC experiments. Sample conditions were 0.5 mM protein, 10 mM Tris pH 7.1, 100 mM NaCl, 4 mM CaCl2, 4 mM DTT, 2 mM TCEP, 100 μM NaN_3_, 5% glycerol, 10% D_2_O.

Data for ^13^C/^15^N labeled human thiopurine methyl transferase (TPMT)*1 was recorded at 800 MHz, 25 °C and consisted of TROSY versions of HSQC, HNCA, HN(CO)CA, HN(CA)CB, CBCA(CO)NH, HNCO and HN(CA)CO experiments. Due to poor sensitivity duplicate data sets were recorded and co-added. Sample conditions were 0.5 mM protein, 20 mM KP_i_ pH 7.3, 75 mM NaCl, 0.5 mM TCEP, 200 μM NaN_3_, 2% glycerol, 10% D_2_O. The protein construct was truncated to omit the 15 N-terminal residues.

Data for ^13^C/^15^N/^2^H labeled S677/680A, D754A mutant of murine ephrin receptor type-B2 juxtamembrane segment and kinase domain (EphB2 JMS-KD) were performed at 600 MHz, 25°C and consisted of TROSY versions of HSQC, HNCA, HN(CO)CA, HN(CA)CB, HN(COCA)CB, HN(CA)CO and HNCO experiments. In applicable cases deuterium decoupling was applied. Sample conditions were 0.6 mM protein, 20 mM HEPES pH 7.2, 150 mM NaCl, 200 μM NaN_3_, 2% glycerol, 10% D_2_O.
